# Oxidative Stress and Exhaled Breath Analysis: A Promising Tool for Detection of Lung Cancer

**DOI:** 10.3390/cancers2010032

**Published:** 2010-02-08

**Authors:** Hiang Ping Chan, Craig Lewis, Paul S. Thomas

**Affiliations:** 1 Faculty of Medicine, University of New South Wales, Sydney, NSW 2052, Australia; 2 Department of Respiratory Medicine, Prince of Wales Hospital, Randwick, NSW 2031, Australia; E-Mail: z3134017@student.unsw.edu.au (H.P.C.); 3 Department of Medical Oncology, Prince of Wales Hospital, Randwick, NSW 2031, Australia; E-Mail: craig.lewis@sesiahs.health.nsw.gov.au (C.L.)

**Keywords:** exhaled breath analysis, lung cancer, oxidative stress

## Abstract

Lung cancer is one of the few neoplasia in which the principal aetiology is known, with cigarette smoke donating a considerable oxidative burden to the lungs. This may be part of the aetiology of lung cancer, but the neoplastic process is also associated with increased oxidative stress. Nonetheless, it is difficult to study the mechanisms behind the induction of lung cancer in smokers, but newer techniques of breath analysis targeting markers of oxidative stress and anti-oxidant capacity show promise in unravelling some of the pathways. This review highlights recent developments in the assessment of oxidative stress by non-invasive methods of breath analysis which are becoming powerful research techniques with possible clinical applications.

## 1. Introduction

Lung cancer is the leading cause of cancer mortality, accounting for 28.3% of cancer deaths in the United States in 2009 [1]. It is also the most common malignancy in the American population, with an estimated burden of 14.8% of all new cases [1]. Despite advances in therapeutic strategies, the five-year survival rate remains dismal at 16% [1]. This can be attributed to the lack of a validated method of screening for early detection of lung cancer given that current evidence does not support lung cancer screening using chest radiography, sputum cytology, or low-dose computed tomography as studies investigating the use of these techniques as screening tools have not reported survival benefits [2]. Therefore, patients with lung cancer presently present at a later stage resulting in a poorer prognosis given the limited and suboptimal therapeutic options for advanced disease [3]. It is imperative that a new validated tool for screening be found as five-year survival rate can be as high as 60–80% if the lung cancer is discovered at an early stage and hence amenable for surgical resection [4,5].

**Figure 1:**
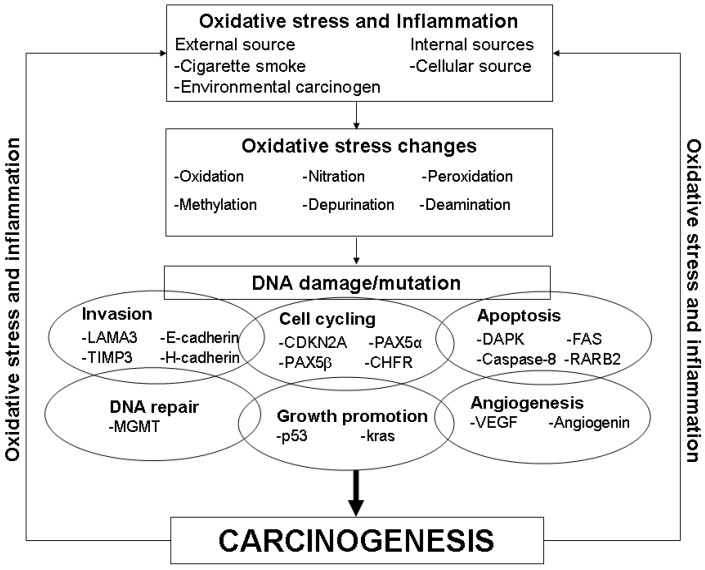
Oxidative stress and its role in carcinogenesis.

Current understanding of the aetiology of lung cancer suggest that oxidative stress is implicated in its pathogenesis with both internal (e.g., cellular production of free radicals) and external (e.g., cigarette smoke and environmental carcinogens) sources, with cigarette smoke conferring a significant amount of oxidative stress and hence the main risk factor for the development of lung cancer [6,7,8,9]. Oxidative stress results in DNA damage (e.g. DNA adduct formation, DNA double strand breaks, DNA mutation) via numerous pathways ranging from oxidation, nitration, depurination, lipid peroxidation, methylation and deamination (Figure 1) [6,8]. Damage to DNA can result in a myriad of changes affecting pathways such as cell cycling, growth promotion, DNA repair, apoptosis, invasion and angiogenesis, thereby inducing the process of carcinogenesis (Figure 1) [3]. However, it is important to note that the neoplastic process in itself also induces a pro-inflammatory state and hence contributes to the oxidative stress burden. Nonetheless, oxidative stress has been strongly implicated in the pathogenesis of lung cancer and hence provides an excellent target for various techniques aimed at early detection of lung cancer. In this review, we will be discussing the use of various methods of exhaled breath analysis in detecting oxidative stress and its associated effects, as well as its potential as a screening tool for the early detection of lung cancer.

## 2. Exhaled Breath Analysis

Exhaled breath analysis is a promising and non-invasive approach for the detection of biomarkers associated with oxidative stress in the respiratory tract and has previously being shown to measure differences in levels of oxidative stress or inflammatory markers in patients with various respiratory conditions such as asthma, chronic obstructive pulmonary disease and bronchiectasis [10,11,12]. There has been an increasing interest in the use of exhaled breath analysis for studies into the early detection of lung cancer as seen in the burgeoning number of works in recent years. Prior to the turn of the new millennium, most of studies involving exhaled breath analysis were focused on other respiratory conditions. Various methods of exhaled breath analysis exist and are primarily subdivided into: (1) gaseous phase analysis or (2) liquid phase analysis, otherwise known as exhaled breath condensate. 

### 2.1. Gaseous Phase Analysis

Gaseous phase analysis allows for either online or offline measurement of the markers of interest using a variety of equipment. In online measurement, patients breathe directly into the machine; whereas in offline measurements, patients breathe into a gas-impermeable bag for breath collection prior to analysis by the machine. Both gaseous and liquid phase analyses sample the respiratory tract for various markers of interest. For the gaseous phase analysis, it is thought that markers can be formed in both the upper and lower respiratory tract and diffuse into the lumen down a concentration gradient, thereby allowing their measurement in the exhaled breath [13]. The two main groups of markers measured are exhaled nitric oxide and volatile organic compounds.

#### 2.1.1. Exhaled Nitric Oxide

Nitric oxide is a free radical that is directly measurable in the gaseous phase of exhaled breath analysis but not in the aqueous phase as it reacts with reactive oxygen species to form peroxynitrite which is then broken down to nitrite and nitrate [14].

Exhaled nitric oxide can be measured using a chemiluminscence analyser and the American Thoracic Society and European Respiratory Society have made recommendations to standardise both the online and offline measurement of exhaled nitric oxide [13]. In a study by Liu *et al.*, the level of exhaled nitric oxide was significantly higher in lung cancer patients when compared with controls (16.9 ± 0.9 p.p.b. versus 6.0 ± 0.5 p.p.b., p < 0.001) suggesting that airway inflammation occurs in association with the neoplastic process [15].

#### 2.1.2. Volatile Organic Compounds (VOCs)

VOCs are markers of oxidative stress derived from lipid peroxidation of polyunsaturated fatty acids and other reactions of reactive oxygen species [16,17,18]. VOCs can be detected by several methods:

(a) Gas chromatography/mass spectrometry analysis (GC/MS)

There have been several studies demonstrating the use of GC/MS in the detection of specific VOCs in the exhaled breath of lung cancer patients and Gordon *et al.* demonstrated that with a profile of three VOCs (acetone, methyl ethyl ketone and *n*-propanol) 93% of the 29 subjects (12 lung cancer patients and 17 controls) could be correctly classified [19].

Phillips *et al.* showed that with a profile of 22 VOCs, comprising predominately of alkanes, alkane derivatives and benzene derivatives, a sensitivity of 71.7% and specificity of 66.7% for detecting lung cancer were achieved [20]. In another study, Phillips *et al.* demonstrated a sensitivity of 85.1% and a specificity of 80.5% with a profile of nine VOCs comprising of C_4_–C_20_ alkanes and monomethylated alkanes [21]. A study by Poli *et al.* consisting of non-small cell lung cancer (NSCLC) patients, COPD patients, smokers and non-smokers demonstrated that 82.5% of all subjects were classified correctly while the sensitivity and specificity for detection of NSCLC was 72.2% and 93.6% respectively with the use of a profile 13 VOCs- isoprene, 2-methylpentane, pentane, ethylbenzene, trimethylbenzene, toluene, benzene, heptane, decane, styrene, octane and pentamethylheptane [22].

Likewise, Ligor *et al.* using a smaller profile of eight VOCs (1-propanol, 2-butanone, 3-butyn-2-ol, benzaldehyde, 2-methylpentane, 3-methylpentane, *n*-pentane and *n*-hexane) demonstrated a sensitivity of 51% and a specificity of 100% [23].

More recently, 1-butanol and 3-hydroxy-2-butanone were found at significantly higher concentrations in patients with NSCLC when compared with controls [24]. Furthermore, 1-butanol was shown to have a sensitivity of 95.3% and a specificity of 85.4% while 3-hydroxy-2-butanone had a sensitivity of 93.0% and a specificity of 92.7% [24]. 

Thus, VOCs as indicators of peroxidation, show considerable promise in terms of detecting the oxidative stress associated with lung cancer. 

(b) Colorimetric sensor array

Mazzone *et al.* had subjects breathe directly into a device that channels their breath across the colorimetric sensor array. A prediction model was derived from observations of 70% of the subjects prior to testing on the remaining 30% and it was shown that this model had a sensitivity of 73.3% and a specificity of 72.4% [25].

(c) Ion mobility spectrometry (IMS)

The working principles of IMS are: (1) separation of analytes in gas; (2) ionisation of analytes via radiation; (3) further separation in an electric field; and (4) visualisation in a three-dimensional IMS chromatogram [26]. Westhoff *et al.* were able to classify and differentiate patients with lung cancer from healthy control with an error rate of zero through the use of a combination of 23 peak regions within the IMS chromatogram [26].

(d) Gold nanoparticles sensor array

There have been two studies looking into the use of gold nanoparticles sensor array. One study demonstrated that based on this sensor array and the use of a multidimensional dataset model, the breath of NSCLC patients could be distinguished from controls as the clusters do not overlap [27]. Furthermore, they also identified 15 VOCs that occur exclusively in NSCLC patients and not the controls [27]. Another study by Peng *et al.* also demonstrated similar finding with the breath of NSCLC patients and controls being distinguished using a multidimensional dataset model [28].

(e) Electronic nose

The electronic nose (eNose) is a technology based on sensor arrays technology that response to and characterise stereochemical properties of individual molecules such as VOCs prior to applying statistical or structural algorithm to distinguish the volatile patterns [29]. This will then allow for a specific “smellprint” of the exhaled breath of lung cancer patients to be created and contrasted against those of healthy controls. Di Natale *et al.* demonstrated that by applying partial least squares-discriminant analysis (PLS-DA) on the data derived from the eNose, the sensitivity in detecting lung cancer was 100% and the specificity was 94% [30]. These data were supported by other studies which applied principal components and canonic discriminant analysis on the sensor data and found that an eNose had a sensitivity of 71.4% and a specificity of 91.9% in detecting lung cancer [29] and could differentiate patients with NSCLC from patients with COPD and healthy controls [31].

A further study also utilized PLS-DA with an eNose and demonstrated a sensitivity of 85% and a specificity of 100% in detecting lung cancer patients amongst a group of healthy control, as well as a sensitivity of 92.8% in detecting lung cancer patients from amongst patients with other respiratory conditions [32]. Finally, Tran *et al.* demonstrated significant differences in three parameters of the “smellprints” of lung cancer patients when compared with the control group which consisted of non-smokers, smokers, ex-smokers and patients with respiratory conditions [33].

The eNose technology does appear to have very encouraging results from the various studies performed. It is, however, important to note that some of the subjects in these studies were diagnosed with advanced disease. Hence, it is imperative that future studies should be targeted at diagnosing patients who are in the early stages of the disease where they are still amenable for curative surgery.

### 2.2. Liquid Phase Analysis/Exhaled Breath Condensate

Exhaled breath condensate (EBC) allows for a convenient and non-invasive method of sampling the respiratory tract [34]. EBC has several advantages: (1) it is easily performed and the volume collected is not affected by age or gender; (2) it allows for repeated measurements in patients with severe diseases; (3) it is an acceptable method of airway sampling for patients as it only involves tidal breathing; (4) it does not induce or influence inflammatory changes in the airway unlike other sampling methods such as bronchoalveolar lavage or induced sputum; and (5) it has been previously validated for use in sampling various constituents of the exhaled breath such as inflammatory markers or DNA in patients with various respiratory conditions [35,36,37].

EBC can be obtained either by the use of custom-made collection devices with cooling apparatus consisting of either ice, dry ice or liquid nitrogen or commercially available devices such as the ECoScreen (Erich JAEGER, GmbH, Hoechberg, Germany) or RTube (Respiratory Research, Charlottesville, VA, USA) [38,39]. The EBC collected contains mainly water (>99.99%), but it also contains aerosol particles from the lower respiratory tract [40]. However as the condensate measurements may reflect the different markers derived from each part of the oro-respiratory tract, there is uncertainty regarding the exact proportion each compartment contributes and this is deemed to be one drawback of EBC [17]. Another drawback of EBC has been its reproducibility as a result of variability in the equipment used, collection methods and the assays employed for detection of biomarkers [35,39,41,42]. Furthermore the variable dilution of respiratory droplets may also contribute to variability in measurements of most parameters of EBC. This has prompted researchers to seek suitable dilution factors through the use of markers such as exhaled volume, conductance of lyophilised samples, exhaled ions, urea or protein concentrations, to correct for and standardise measurements [34,42]. To counteract these problems, the American Thoracic Society and the European Respiratory Society have developed a comprehensive list of recommendations such as the use of a unidirectional valve and salivary trap to standardize the collection process for EBC [42].

In recent years, there have been an increasing number of studies looking into EBC as a potential screening tool for lung cancer. In this section, we will discuss markers of oxidative stress and other measurable parameters associated with oxidative stress that are found in the EBC of lung cancer patients.

(a) Oxidative stress and antioxidant capacity

Chan *et al.* demonstrated that patients with NSCLC had significantly higher levels of hydrogen peroxide and significantly lower levels of antioxidant capacity when compared with smokers [43]. These findings suggest that disequilibrium between levels of oxidants and antioxidants exists in lung cancer and this leads to increased oxidative stress. This provides further evidence that oxidative stress is implicated in the pathogenesis of lung cancer and that biomarkers associated with this process can be readily assayed in the EBC collected from these patients.

There have been several studies looking into lipid peroxidation which is an index of free radical activity. Results have, however, been mixed. In the abstract of a Russian language manuscript, Khyshiktyev *et al.* have reported, without specifying the actual marker, that levels of lipid peroxidation were lower in EBC of patients with lung cancer compared with controls [44]. In two recent studies by Chan *et al.* and Dalaveris *et al.*, there was, however, no significant difference in the levels of 8-isoprostance between lung cancer patients and controls [43,45]. This suggests that 8-isoprostane may not be useful as a biomarker in the EBC of lung cancer patients and that other products of lipid peroxidation should be studied.

(b) Associated changes- DNA damage/mutation

As discussed in the introductory section, oxidative stress can result in DNA damage or mutations leading to various changes in cell cycling, growth promotion, DNA repair, apoptosis, invasion and angiogenesis (Figure 1). Therefore, such changes can possibly be detected and studied in the EBC of lung cancer patients.

Gessner *et al.* demonstrated p53 mutations in the EBC of 36.4% of study lung cancer patients but none in controls [46]. The authors suggest that EBC may thus have a role in detecting somatic gene mutations in areas of direct tobacco-related DNA damage [46]. They also demonstrated that there were significance differences in the levels of angiogenic markers such as VEGF, bFGF and angiogenin in the EBC of NSCLC patients compared with COPD patients [47]. In addition, Carpagnano *et al.* demonstrated 3p microsatellite alterations in the EBC of 53% of lung cancer patients compared with 13% of healthy controls [48]. They also showed that the number of 3p microsatellite alterations found in the EBC of lung cancer patients exhibits good correlation with patient’s survival [49]. Finally, Han *et al.* showed that gene promoter methylation could be detected in the EBC of lung cancer patients and that CpG at −63 of DAPK promoter and +52 of PAX5beta promoter were significantly associated with lung cancer status [50].

(c) Inflammatory markers (proteins and cytokines)

Various markers of cancer-associated inflammation have been studied. Endothelin-1 was found to be elevated in the EBC of NSCLC patients compared with controls and significantly increased in patients with Stage IV disease compared with patients with Stages I–III disease, suggesting that it may be related to tumour burden [51]. Likewise interleukin-6 was elevated in the EBC of NSCLC patients compared with controls and there was a significant correlation between IL-6 levels and stage of disease [52].

Carpagnano *et al.* demonstrated significantly higher levels of interleukin (IL)-2, leptin and tumour necrosis factor-alpha (TNF-α) in the EBC of lung cancer patients when compared with controls, with levels of these markers being positively correlated with the stage of the disease [53]. These findings have been partially corroborated by a separate study showing that significantly higher levels of TNF-α were present in the EBC of lung cancer patients compared with healthy smokers [45].

Carpagnano *et al.* also demonstrated higher levels of cyclooxygenase-2 and survivin in the EBC of NSCLC patients when compared with healthy smokers and non-smokers [54]. Furthermore, a correlation was also found between levels of COX-2 and survivin and the progression of cancer [54]. These studies appear to indicate that the neoplastic process is associated with inflammation as well as the processes classically implicated in carcinogenesis.

## 3. Conclusions

Currently, some of these methods allow us to detect and quantify the individual molecules (*i.e.*, gaseous phase analysis of exhaled NO and VOCs, and exhaled breath condensate) that may be implicated in the carcinogenesis process, while others allow for a global pattern of markers to be formulated and recognized (*i.e.*, an eNose). This would allow us to have greater insight into the pathogenesis of lung cancer as well as to provide a non-invasive and potentially powerful method of sampling the airways for early detection of lung cancer.

These methods clearly have great potential as a tool for early detection of lung cancer. However, they are currently not employed in clinical practice as several issues need to be addressed. Firstly, there has to be a validated marker or panel of markers to be used in the detection of lung cancer. Secondly, there have been questions raised over the reproducibility of these methods such as exhaled breath condensate where certain biomarkers such as hydrogen peroxide has been shown to have day-day intra-subject coefficient of variation of up to 43% while others such as nitrites, nitrates, leukotrienes, 8-isprostanes have not had any reported data on their day-day coefficient of variation [34,42]. This is potentially a drawback for these methods as they have to be demonstrated to have a high reproducibility in order to be validated as an accurate tool for lung cancer detection. Lastly, there has not been any studies looking into the cost-effectiveness of these methods and this is an aspect that requires more research.

This review has highlighted the role of exhaled breath analysis as a non-invasive and promising tool for detecting oxidative stress changes in the respiratory tract. We acknowledge that oxidative stress may be part of the pathogenesis of lung cancer and/or a response to neoplasia. While it may be difficult to uncover the exact contribution of each component, exhaled breath analysis remains an excellent tool for detecting such changes, and hence with further studies could potentially be used to screen for lung cancer.
